# 169. Prevalence and Associated Patient Characteristics of Multi-Drug Resistant Organisms and Antibiotic Prescribing Patterns in Hospitalized Patients with Community-Acquired Pneumonia

**DOI:** 10.1093/ofid/ofab466.371

**Published:** 2021-12-04

**Authors:** Leah Rodriguez, Kazumi Morita, Elizabeth Tencza, Daniel Mueller

**Affiliations:** 1 Temple University School of Pharmacy, Philadelphia, Pennsylvania; 2 Temple University Hospital, Plymouth Meeting, Pennsylvania

## Abstract

**Background:**

The 2019 Infectious Diseases Society of America/American Thoracic Society community-acquired pneumonia (CAP) guidelines eliminated the term healthcare-associated pneumonia (HCAP), and recommends to guide the use of broad-spectrum antibiotics by locally validating the prevalence and risk factors of multi-drug resistant organisms (MDROs). The objective of this study is to determine the prevalence and associated patient characteristics of MDROs, and to characterize antibiotic prescribing patterns.

**Methods:**

This was a retrospective, cohort study in adult patients hospitalized from 1/1/19 to 12/31/19. Patients were randomly selected from a patient list of diagnosis codes suggestive of pneumonia. We excluded patients with antibiotic therapy < 48 hours, bacterial co-infections from another site, or transferred from another hospital with length of stay >24 hours. Endpoints evaluated include the percentage of MDRO isolated from a respiratory or blood culture collected within 2 days of admission, comparison of patient characteristics associated with MDROs with those who did not, treatment regimen and duration, and rate of overtreatment and undertreatment.

**Results:**

A total of 220 patients were included. Prevalence of overall MDRO, methicillin-resistant *Staphylococcus aureus* (MRSA), and *Pseudomonas aeruginosa* (PSA) was 8%, 3%, and 5%, respectively. Patient characteristics associated with MDROs from are shown in Table 1. Prior MDRO history or recent intravenous (IV) antibiotic exposure during hospitalization was present in 39% of the MDRO cohort. Over half (58%) of the patients were initiated on antibiotics with MRSA and/or PSA coverage. Rate of overtreatment and undertreatment was 89% and 5%, respectively. Mean antibiotic duration was 9 ± 3 days.

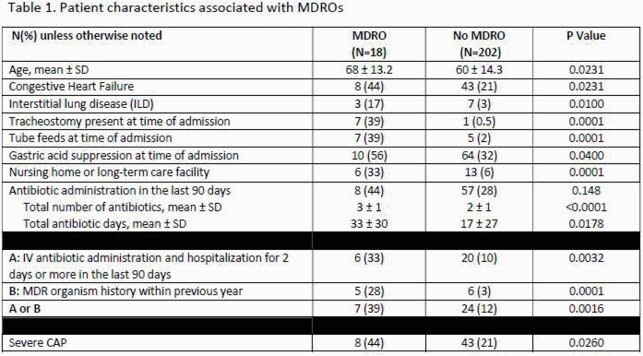

**Conclusion:**

The low prevalence of MDROs, coupled with the high overtreatment and low undertreatment rate suggests most patients hospitalized with CAP at our institution can receive an antibiotic regimen targeting standard CAP pathogens. Antibiotic stewardship intervention to shorten the duration of therapy should be considered. In addition to microbiology history and recent IV antibiotic exposure during hospitalization, further studies are needed to validate other patient characteristics at risk for MDROs.

**Disclosures:**

**All Authors**: No reported disclosures

